# Is there an innate sex-based difference in the manner in which the left ventricle responds to the pressure overload of aortic stenosis?

**DOI:** 10.1186/1532-429X-11-S1-P237

**Published:** 2009-01-28

**Authors:** Saundra Grant, Diane A Vido, Vikas K Rathi, June A Yamrozik, Ronald B Williams, Geetha Rayarao, Mark Doyle, Robert WW Biederman

**Affiliations:** Allegheny General Hospital, The Gerald McGinnis Cardiovascular Institute, Pittsburgh, PA USA

**Keywords:** Aortic Stenosis, Aortic Valve Replacement, SSFP Imaging, Genotypic Alteration, Pressure Afterload

## Introduction

Numerous investigators have suggested that women are particularly prone to develop an exaggerated LV hypertrophy (LVH) response to increased developed pressure as compared to men. This has been best exemplified in both HTN and aortic stenosis (AS) states and has been proposed euphemistically as 'exuberant hypertrophy'. This reference implies that there is an inappropriate physiologic amplification to the typical and necessary hypertrophic response for any given blood pressure/afterload experienced by the LV. Furthermore, it suggests that there is a divergent response at the gene level to explain this phenomenon. The implications for this are vast, potentially calling for distinct sex-based clinical treatment programs. The societal ramifications for differential therapy are immense as our resources are limited. On the other hand, the majority of the inferences of this dichotomized observation have stemmed from 2D echocardiography and consequently may be artifactual if evaluated in a 3D CMR manner.

## Hypothesis

We hypothesize that 'exuberant hypertrophy' is a misnomer and that after aortic valve replacement (AVR) there will be no sex difference in the manner or rate in which LVH regresses following afterload relief.

## Methods

Following initial recruitment of 35 patients for a longitudinal 2-year CMR study, a series of patients who were alive and available were specifically contacted and re-recruited for follow-up up to 4 years from their initial AVR. All patients underwent an additional CMR for evaluation of 3D volumetrics, including LV mass and EF. Using Simpson's rule, contiguous 8 mm slices were acquired via SSFP imaging and underwent LV endocardial and epicardial manual contouring (GE 1.5 T EXCITE, Milwaukee, WI). Pre and post-AVR LV mass and EF were compared and stratified for sex. A 'p' value of <0.05 was considered statistically significant.

## Results

**Figure 1:**
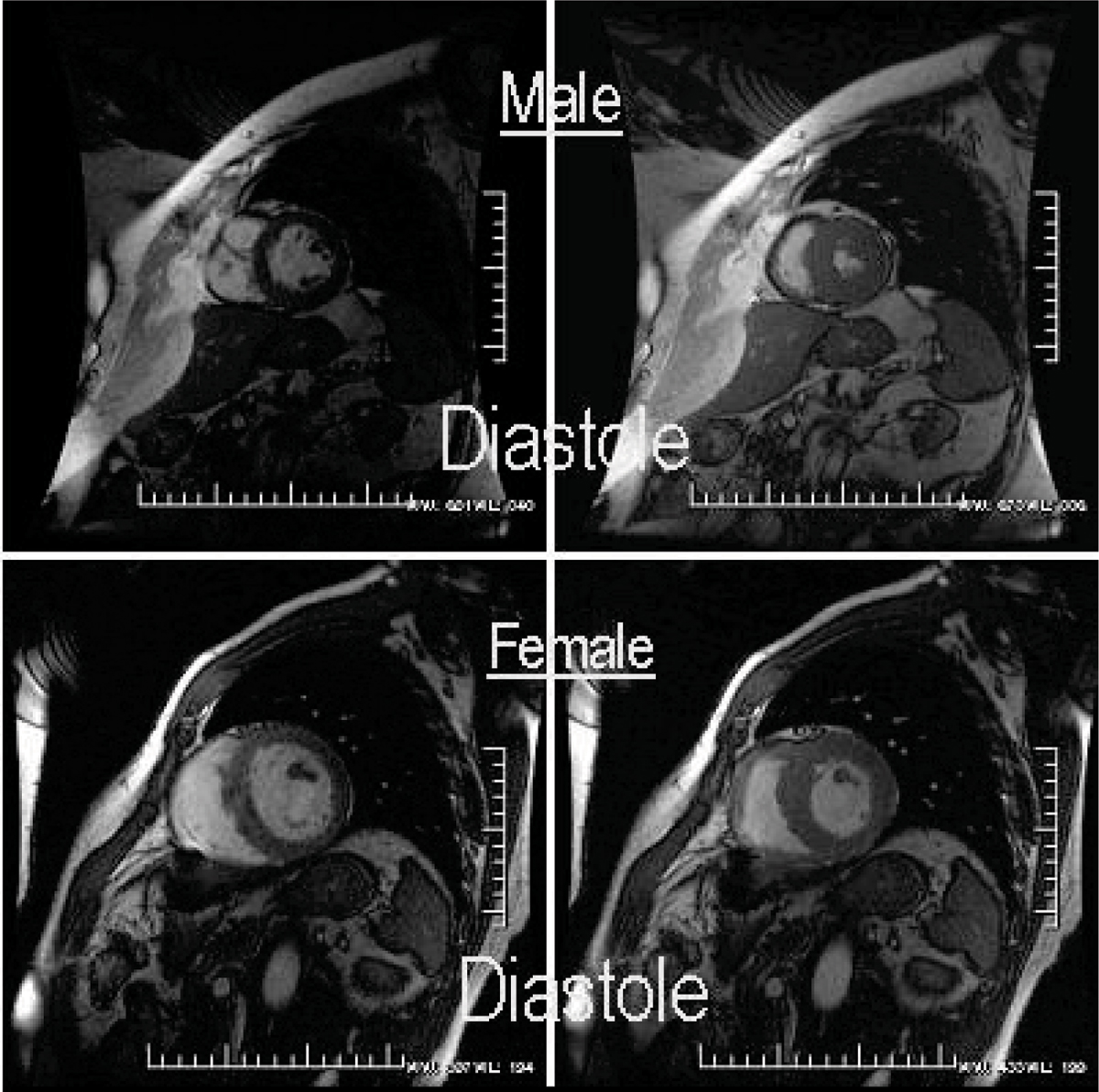
**2D CVMRI images of geometry in A) 65 YO female with a small thick LV and B) 68 YO male with a larger, thinner LV, both with similar mean gradient (52 ± 4 mmHg), BSA (2.1 ± 1), and LVMI corrected for EDV (1.33 ± 0.10) and with similar LVMI**.

Ten (10) patients were available for CMR at 3 ± 1 yrs out to 4 years at follow-up post-AVR and successfully underwent CMR. Women (6; age: 68 ± 14) and men (4; age: 68 ± 10); (p < NS for both) were imaged. Baseline LV mass index was neither increased in females nor different between women and men (75 ± 16 vs. 101 ± 23 g/m^2^, p = NS). However, baseline EF was dissimilar between women and men (67 ± 12 vs. 37 ± 23%, p = NS). Following AVR, in absolute or relative terms, LV mass index regression was not different between the sexes (65 ± 17 vs. 73 ± 11 g/m^2^; representing a proportionate decrease in LV mass of 11 ± 16 vs. 28 ± 17 g/m^2^, p = NS), (Figure 1). Likewise, post-AVR, LVEF was not significantly different (68 ± 7 vs. 61 ± 10%, p = NS, but did markedly improve in the males (1 ± 10 vs. 24 ± 20%, p < 0.05), respectively for women vs. men, again without any absolute change in LV mass index between groups.

## Conclusion

While conceptually intriguing and highly controversial, the sex-based differences of exaggerated LVH formation in response to pressure afterload that are based substantially on 2D echocardiographic acquisitions appear to be *artificial* when based upon a 3D CMR interrogation. Specifically, after recruiting a select group of patients with AS who underwent AVR with up to 4 years of follow-up, more than sufficient time to allow for regression of LV mass to near completion (if it was going to) failed to demonstrate a significant difference as related to women vs. men. Beyond negating the 2D observation by a more sophisticated 3D CMR interrogation, the potential socio-economic repercussions are nullified should a more favorable response to afterload have been found (either at baseline or after AVR). This finding may restore the notion that there are neither phenotypic nor likely genotypic alterations in the LV response to pressure afterload in women vs. men.

